# Are There Reliable Qualitative Individual Difference in Cognition?

**DOI:** 10.5334/joc.131

**Published:** 2021-08-27

**Authors:** Jeffrey N. Rouder, Julia M. Haaf

**Affiliations:** 1 University of California, Irvine, US; 2 University of Amsterdam, NL

**Keywords:** Individual Differences, Cognitive Tasks, Hierarchical Models, Bayesian Inference

## Abstract

In this paper we propose a new set of questions that focus on the direction of effects. In almost all studies the direction is important. For example, in a Stroop task we expect responses to incongruent items to be slower than those to congruent ones, and this direction implies one theoretical explanation. Yet, if congruent words are slowed down relative to incongruent words we would have a completely different theoretical explanation. We ask a ‘does everybody’ question, such as, ‘does every individual show a Stroop effect in the same direction?’ Or, ‘does every individual respond faster to loud tones than soft tones?’ If all individuals truly have effects in the same direction that implicate a common theory, we term the differences among them as quantitative individual differences. Conversely, if all individuals truly have effects in different directions that implicate different theories, we term the differences among them as qualitative individual differences. Here, we provide a users guide to the question of whether individual differences are qualitative or quantitative. We discuss theoretical issues, methodological advances, new software for assessment, and, most importantly, how the question impacts theory development in cognitive science. Our hope is that this mode of analysis is a productive tool in researchers’ toolkits.

## Introduction

At the heart of experimental psychology is the experimental method. By creatively manipulating some aspects of the stimuli or task while controlling for others, experimental psychologists hope to isolate latent mental phenomena. To fully leverage the experimental method, these manipulations may be yoked to within-subject designs where each person acts as their own control. The potential power of the experimental method with within-subject designs seems unrivaled in cognitive psychology.

Although the experimental method yoked with within-subject designs is powerful, the typical questions psychologists ask in such settings strike us as limited. The vast majority of our questions concern hypotheses about population averages rather than about individuals. To see this limitation, consider the case with two conditions where the goal is to assess if there is an effect and, if so, in which direction. For example, the conditions might be the congruent and incongruent conditions of a Stroop task, and the question is whether there is a true effect where the effect is about the population mean, or the average over people. The concept of *average over all people*, however, is quite abstract; psychology, in contrast, is about mental processing within individuals. We argue that the experimental method with within-subject designs allows researchers to do better. With expanded analyses, which we provide here, they can treat processing as specific to individuals. Moreover, they can ask about the range of processing across the population.

To develop these questions about individuals we start with an observation that theories often describe ordinal rather than metric constraint (Haaf et al., 2019). In the Stroop case, for example, there are no theories that predict whether the effect should be 4 ms, 40 ms, or 400 ms. All theories we are aware of predict that congruent items are responded to more quickly than incongruent items. Yet, the degree of speed up is either unspecified or a free parameter in a model. And so, we typically ask ordinal questions of group averages: are these averages truly positive, negative, or null?

**Figure 1:**
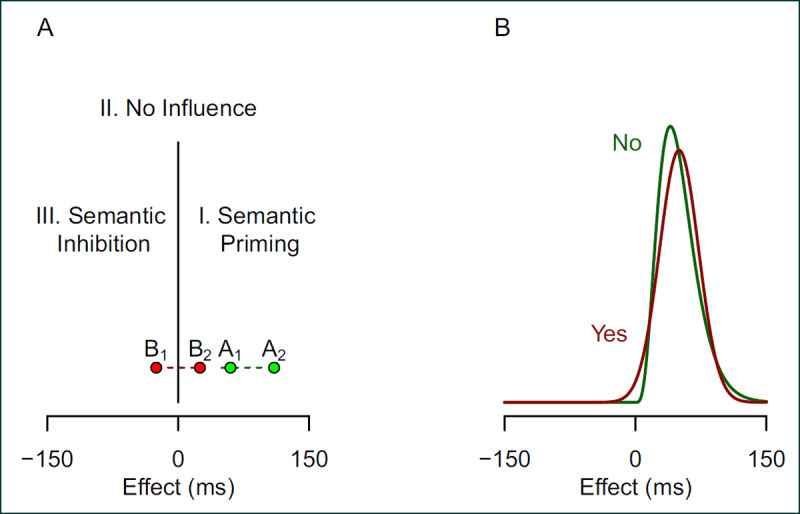
Qualitatively different regions of effects. **A.** The three regions are positive, zero, and negative effects, and they correspond to three distinct classes of theories. **B.** The distributions of individual effects can either be in one region or span several regions. The distribution labeled “No”, which has no qualitative individual differences, is constrained to the positive region. The distribution labeled “Yes” spans all three regions.

Given the coarse state of theory, we argue that these ordinal questions make sense. Let’s define the effect as the difference between conditions and positive values as those that denote a usual or expected direction. In the Stroop task, positive effects denote the case when responses to congruent items are faster than those to incongruent items. ***Figure 1A*** shows three regions that correspond to three different classes of theories. The first region, the positive region, corresponds to usual theories where reading is quick, automatic, and biases the response toward the word identity. We can call these *semantic priming* theories. The second region, comprised of the null point, corresponds to theories where the semantic nature of the word has no influence on the response. This could occur because reading is slower than color naming, or if faster, does not bias responses. The third region, the negative region, corresponds to theories where the semantic meaning of the word inhibits the same color response. For the purpose of this paper, we could call these theories *semantic inhibition* theories, though we note that there is no evidence anywhere in the literature of a semantic inhibition Stroop effect (MacLeod, 1991). The usual question is where does the true population average effect fall—is it in the positive, null, or negative region. And this usual question makes some sense as these are the only three regions that have theoretical importance.

Group- or population-level averages are fine as a start, but are inherently limited. Rather than ask an *on average* question, we advocate a new question: *Are there qualitative individual differences?* Does every individual have a true effect in the same theoretically meaningful region? Or do some people have true effects in one region and others in another. The question is not about the average—it is about the distribution of ordinal properties across individuals.

To expand on the question, consider the two hypothetical individuals labeled *A*_1_ and *A*_2_ in ***Figure 1***. These individuals have true effects in the same region—both are described by a semantic-priming theory. The 50 ms difference in their true Stroop effect scores, while quantitatively important, is not a qualitative difference. We can contrast this individual difference configuration to that for individuals *B*_1_ and *B*_2_. These two hypothetical individuals are qualitatively different— *B*_2_ is concordant with the semantic-priming theory while *B*_1_ is concordant with semantic-inhibition theory. This qualitative difference holds even though the difference between *B*_2_ and *B*_1_ is the same 50 ms difference as between *A*_2_ and *A*_1_.

***Figure 1B*** shows two cases, both of which correspond to a positive population-average effect. The two distributions are that of individuals’ true effects, and they each have the same mean and variance. For the distribution labeled *No*, all individual true effects are positive, and for all individuals, the semantic priming theory hold. There are *no* theoretically meaningful, qualitative differences across people. For the distribution labeled *Yes*, the majority of individuals have positive effects and a minority have negative effects. Because these are true values, the semantic priming theory holds for some people and the semantic inhibition theory holds for others.

## A Users Guide To Qualitative and Quantitative Individual Differences

The above distinction between qualitative and quantitative individual differences strikes us as foundational. If there are no qualitative individual differences, then the phenomenon under study is seemingly robust across individuals. The theoretical account should be relatively simple, perhaps reflecting automatic or obligatory responses that may be biologically hardwired. If there are qualitative individual differences, then phenomenon under study is rich and complex. Theories would need to be flexible to account for such diversity, and theories that stipulated multiple processes, pathways or strategies may be attractive. Moreover, questions of individual differences would come to the forefront: *Who are these people with negative or null scores? What does non-positivity covary with?*

The goal of this paper is to serve as a *users guide* to qualitative and quantitative individual differences. Our hope is that this distinction is regularly considered. To help speed adoption, we cover a range of issues here. In the next section we cover two outstanding theoretical concerns: whether qualitative individual differences always exist and whether the presence of qualitative individual differences depends on the framing of the question. In the following sections we review the methodological advances for assessing whether there are qualitative individual differences. These advances are vested in the comparison of hierarchical models as developed in Haaf & Rouder (2017). Here we explain in tutorial fashion how the models are specified and compared. We provide an R function that automates the comparison for control-vs-treatment designs, and review how to use this function.

One important question that is this: *How can the field advance by considerating qualitative and quantitative individual differences?* Part of the motivation for writing this paper to this readership is to query your opinions. Does the distinction speak to you and your work? In this paper, we highlight the utility of this distinction by considering several classes of tasks.

## Theoretical Considerations

Our hope is that the answer to the question, *are there qualitative individual differences*, is informative: If the answer is affirmative in a research domain—that is, if some people have truly negative or null effects while others have truly positive effects, then, theoretical accounts would need to be sufficiently complex to account for this diversity. Alternatively, if there are no qualitative individual differences, then the phenomenon under study is seemingly robust across individuals and the theoretical accounts should be streamlined. Before we proceed, there are two outstanding theoretical issues. The first is whether the question itself makes any sense; the second is whether the presence of qualitative differences depends on the framing of the question and the knowledge of the researcher. Indeed it does, and to account for this fact, we introduce the concept of reducible and irreducible qualitative individual differences. We take these two issues in turn.

### Does The Qualitative Individual-Difference Question Make Sense?

One might ask *a priori* if the qualitative individual-difference question makes much sense. We find that most researchers tend to believe that the human condition is so diverse that there must be people who deviate in all behaviors. Indeed, these types of deviations are so unfathomable at times that they make for compelling reality TV shows.^1^ Therefore, it may seem reasonable to stake out a position that there must be some person that is truly qualitatively different regardless of the task. Accordingly, in the Stroop case, there must be some person who truly responds faster to incongruent items than congruent ones. We call this position where there must be qualitative individual differences the *arbitrary behavioral diversity* hypothesis.

Yet, we are not sure this position of arbitrary behavioral diversity is helpful. First, we note that there are scientific invariances and regularities. Consider the startle reflex. It is known that the startle reflex to a loud noise burst increases with intensity of that burst (Blumenthal & Berg, 1986). We may ask whether all (conscious) people have a larger startle reflex to loud bursts than to soft ones. Assuredly, the answer is yes. Indeed, much is known about the nuclei and synaptic connections that comprise startle (Davis, 2006), and it is hard to see how such a primitive, subcortical reflex can reverse across people much less across species. Simply put, we cannot conceive that someone truly startles more to softer noises than louder ones.

For the sake of argument, let’s probe the arbitrary behavioral diversity hypothesis further. Suppose we believe that there must be some people who startle to soft rather than loud stimuli. And if these people are exceedingly rare, we may miss them in our studies. And if we miss them, then our corresponding conclusion that there are no qualitative individual differences may be in error. Yet, we are not so worried. Having a theory that works for all but a few exceedingly rare people would be quite an achievement.

We should also address the opposite *a priori* belief that there may be no diversity in behavior. After all, it may be difficult to think of a task where people differ qualitatively. Yet, there is one obvious counter example—laterality. Consider a task where individuals throw a ball with their left and right hands, and where the measure of laterality is how much further an individual throws the ball with her right hand than with her left hand. Most individuals, the right-handed individuals, have a true positive score on this measure. But around 9% of the population, the left-handed individuals, may have a true negative score (Coren, 1993). Handedness admits qualitative individual differences.

### Reducible and Irreducible Qualitative Individual Differences

There are stimuli that are perceived qualitatively differently by individuals. Two recent famous cases are the *The Dress* (Wallisch, 2017) and the Yanni/Laurel audio clip (Bosker, 2018).^2^ The Dress is perceived by some people as being black and blue striped, and by others as being white and gold striped. The audio clip is perceived by some people as saying “Yanni” and by others as saying “Laurel.”^3^ The explanation of the dress phenomena is that the stimulus is ambiguous with regard to color, and individual differences are a function of the perception of the background illumination. Some people tend to view the dress as being naturally illuminated whereas others as being artificially illuminated, and these assumptions about the nature of the background lighting influence the color perception of the dress (Wallisch, 2017). The perception of the audio clip is influenced by how sensitive people are to the low-frequency register. The clip is designed such that the high-frequency information is more compatible with the “Yanni” interpretation while the lower-frequency information is more compatible with the “Laurel” interpretation. Differences in sensitivity to different frequency ranges result in the qualitative differences in interpretation (Bosker, 2018).

The examples above with The Dress and the Yanni/Laurel sound clip highlight a pathway for understanding qualitative individual differences. For the audio clip, the presence of qualitative individual differences comes about because the stimulus is inherently multivariate and different dimensions of the stimulus are simultaneously analyzed. When these dimensions are in conflict, small differences in receptive sensitivity, say the sensitivity to information in higher auditory frequencies, may have large effects on perception. Here, we can reconcile the differences. It is not so much that people differ in how they encode, integrate, and analyze auditory information or how they convert auditory information to semantic meaning. Instead, the difference may be reduced to rather low-level differences in sensory systems that do not implicate higher-order cognition.

The theme that qualitative individual differences may not implicate large-scale cognitive differences, but rather more mundane differences that only manifest in carefully controlled cases, should be seen as desirable. An example of this type of understanding may be seen in the cross-race face-recognition effect. Here, people study photographs of faces for a subsequent old-new recognition memory test. People are better at recognizing faces from their own race than they are from other races (Meissner & Brigham, 2001). Conditional on a particular stimulus, there are qualitative individual differences. For example, Black people may better identify Black faces than White faces while White people may better identify White faces than Black faces. Yet, these qualitative individual differences are reconcilable. Simply put, every individual may have better recognition with ingroup faces (Bernstein, Young, & Hugenberg, 2007). The ability to reduce qualitative individual differences to quantitative ones represents knowledge that informs theory, and doing so should be a general goal of psychological sciences. Hence, a necessary condition of meeting this goal is to understand whether there are qualitative or quantitative individual differences in a domain.

## A Stroop Example

**Figure 2:**
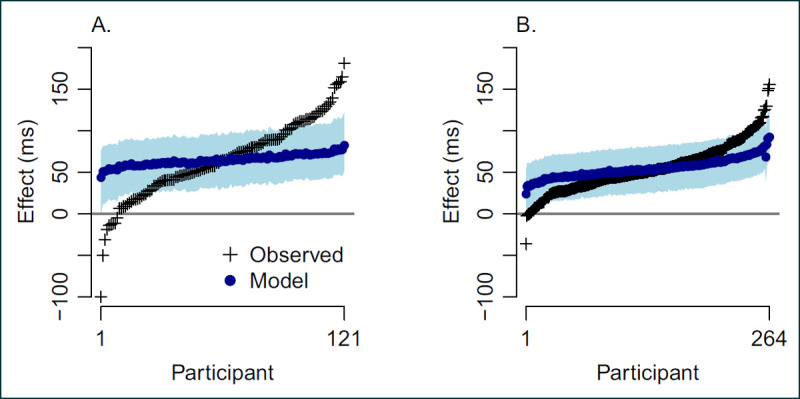
Observed and model-estimated effects. The observed effects are shown as crosses, and the variability of these estimates reflects both trial noise and true variability across people. The model-estimated effects are shown as circles, and they account for trial noise reflecting only true variability across people. **A.** Stroop-effect data from Von Bastian et al. (2015). **B.** Stroop-effect data from Rey-Mermet et al. (2018).

In this section, we provide an example where we ask if there are qualitative individual differences in the Stroop task. ***Figure 2A*** provides a graphical representation of the observed individual variability in a Stroop task from Von Bastian, Souza, & Gade (2015). Each small cross shows the mean difference between the incongruent and congruent conditions for each person. We denote this difference as the *observed effect*, and in ***Figure 2A***, the observed effects are ordered from most negative to most positive. There is quite a bit of individual variation, and indeed, nine of the observed effects are negative indicating the possibility of true qualitative individual differences. ***Figure 2B*** show the same plot for Stroop data from Rey-Mermet, Gade, & Oberauer (2018). Here, only four of the 264 participant have a negative observed effect.

### True vs. Observed Effects

We note the difference between observed individual effects, like the crosses in ***Figure 2***, and hypothetical true effects. True effects are those that occur if we had an unlimited number of trials per person per condition. Yet, we only observe data from a limited number of trials, and the observed effects are perturbed from true effects by sample noise. Fortunately, we can make an educated guess about the effects of this noise as follows: Rouder et al. (2019b) report that the trial-level standard deviation in Stroop and similar tasks is about 200 ms. Therefore, if we had 100 trials per person in a condition, we could reasonable expect to know an individuals’ true mean RT in one condition with a standard error of 20 ms (standard error is the standard deviation divided by the root of sample size). The effect is the differences between condition means, and hence, an additional factor of √2 is required yielding a standard error on the effect of 28 ms. Thus, if there were no individual differences at all, we would expect 28 ms of variability across individuals’ observed effects. Given that average effects are on the order of 50 ms or so, it becomes clear that we should expect some participants to have negative observed values just from trial noise. The upshot is that if we wish to assess qualitative individual differences, we cannot use sample effects. Instead, it is critical to use a model to separate trial noise from true individual differences.

Fortunately, hierarchical models are perfect for separating trial noise from true individual differences. Here, we briefly review how they work in this context. A more complete treatment is provided in Haaf & Rouder (2017).

### Accounting For Trial Noise

Separating true effects from sample noise is at the heart of modern hierarchical modeling. To understand the multiple layers of variability we need a bit of notation. Let *Y_ijk_* be an observation, say a response or a response time. Let the subscript *i* denote individuals, and if there are *I* people in an experiment, then *i* = 1, …, *I* refers to any one of them. Let the subscript *j* refer to conditions, and for our simple example, let *j* = 1 refer to congruent condition and *j* = 2 refer to the incongruent condition. Let the subscript *k* refer to the replicate for the person and condition. In most applications participants perform several trials in each of the two conditions. In general, *k* = 1, …, *K_ij_* where *K_ij_* is the number of replicates for the *i*th person in the *j*th condition. We start with a rather ordinary linear model on response times:

1 {Y_{ijk}} \sim {\rm{Normal}}\left( {{\alpha _i} + {x_j}{\theta _i},{\sigma ^2}} \right),

Here, *α_i_* serves as an intercept, and it is the mean response time for the *i*th person in the congruent condition. The term *x_j_* indicates which condition the response is from. It is given as *x_j_* = 0 if the condition is congruent and *x_j_* = 1 if it is incongruent. This setup implies that *θ_i_* is the effect of condition. Both *α_i_* and *θ_i_* are individual true scores. They describe characteristics of the individual if we ran an experiment with infinitely many trials. Our target is *θ_i_*, the true Stroop effect for each person.

One goal is to estimate each individuals’ *θ_i_*. Here, it makes sense to treat these effects as random effects:

2 {\theta _i} \sim {\rm{Normal}}\left( {{\mu _\theta },\sigma _\theta ^2} \right),

where *μ*_θ_ is the population mean across people and \sigma _\theta ^2is the corresponding population variance of true effects. ***Figure 2*** shows hierarchical model estimates—the filled points are the best point estimates (the posterior means) and the shaded area is the degree of uncertainty (95% credible intervals).^4^ As can be seen, model estimates are far more moderate than the observed effects. The reason is the aforementioned separation between sample noise and true variability. Observed effects reflect both true individual variation and trial noise; model-based estimates reflect only true individual variation without trial noise. This behavior where hierarchical models provide more moderate estimates is a general property and highly advantageous (Davis-Stober, Dana, & Rouder, 2018; Efron & Morris, 1977; Rouder & Haaf, 2019).

### Formulating Constraint

After applying the model, it seems likely that everyone does indeed exhibit a positive Stroop effect in both data sets. There may be no qualitative differences. Yet, although ***Figure 2*** provides a helpful graphical view of true effects, it does not directly answer the question of whether there are qualitative individual differences. To do so, we use model comparison. Here we consider three models—one for the presence of qualitative individual differences, one for the presence of only quantitative individual differences, and one of the absence of any individual differences. We have already seen the model that allows for qualitative individual differences—it is comprised of Equations (1) and (2). We call this the *unconstrained* model because *θ_i_* may take on any value including negative ones. To model the absence of qualitative individual differences, we restrict *θ_i_* > 0. This restriction is implemented in the following model:

3 {\theta _i} \sim {\rm{Norma}}{{\rm{l}}_ + }\left( {{\mu _\theta },\sigma _\theta ^2} \right),

where the normal distribution is now truncated below at zero and forces all values of *θ_i_* to be positive. We call this the *positive model*.

**Figure 3:**
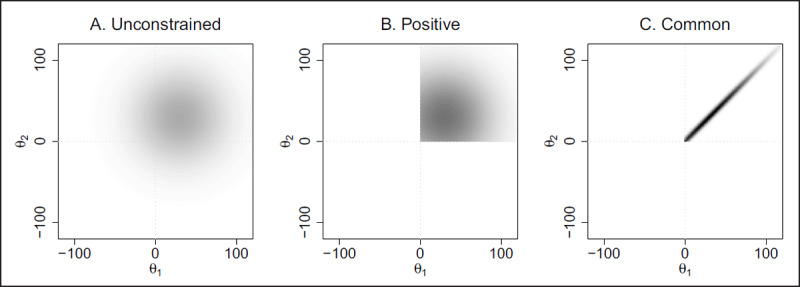
The three models are shown as bivariate distributions across two individuals’ true values (denoted *θ*_1_ and *θ*_2_). Darker areas show greater concentration of density.

It may seem like the models are very similar. In fact, they could be quite different. ***Figure 3*** shows a comparison for two participants. In ***Figure 3A*** shows the unconstrained case where *μ*_θ_ = 30 ms and *σ*_θ_ = 40 ms. ***Figure 3B*** shows the case with positive constraint. As can be seen, the distribution is confined to the upper-right quadrant. Imagine if there were 100 participants. The probability that all 100 have true positive values is quite small under the unconstrained model yet exactly 1.0 under the positive one.

There is an even more constrained model that may be considered—there are no individual differences whatsoever. We have previously called this the *common-effect model* (Haaf & Rouder, 2017), and it is shown in ***Figure 3C***. This model captures the notion that individual differences are so small that a model without them may describe the data better than models with them.

### Comparing Models

There are three models: a. The unconstrained model which captures qualitative individual differences; b. the positive model which captures quantitative individual differences; and c. the common-effect model which captures a lack of individual differences. The next question is how to compare these models in light of data. The main problem here is that the positive model is a subset of the unconstrained model another. Yet, both models have the same number of parameters. Consequently, the comparison is difficult from a classical perspective (see Silvapulle & Sen, 2011). Yet, inference is surprisingly convenient from a Bayesian perspective (Gelfand, Smith, & Lee, 1992). And the computation of Bayes factors—the strength of evidence from data for one model relative to another—is relatively straightforward for the inequality constraints in the positive model (Hoijtink, Klugkist, & Boelen, 2008; Klugkist & Hoijtink, 2007; Klugkist, Laudy, & Hoijtink, 2005). In our previous work, we provide the development of Bayes factor solutions (Haaf & Rouder, 2017, 2019). We have curated this development into one easy-to-use R function described next.

## Function quid(): The Qualitative-Individual-Differences Test Function

Here we provide the easy-to-use R function, quid(), (for qualitative individual differences) to perform the assessment. We review the main elements here, and a tutorial is available.^5^

The function quid() runs on any R platform including the R, RStudio, and RStudio Cloud. The first step is installing the function. At the R command prompt, type the following:


        install.packages(c("BayesFactor", "MCMCpack"))
source("https://bit.ly/2ZqGOik")
      

To understand the function, we first start with some example data from Von Bastian et al. (2015), which are shown in ***Figure 2A***. The data are publicly available and can be cleaned and loaded into R with the following code:


        source("https://bit.ly/2SxyKtq")
      

After running the above code the data are loaded as a data frame called stroop. Each row of the data frame corresponds to a single trial for a single participant. The participant identification number is the variable stroop$ID (from 1 to 121), the condition is the variable stroop$cond (values are 1 for congruent and 2 for incongruent), and the response time for each trial in seconds is stored in the variable stroop$rt. These three variables have to be passed to the function quid() to run the analysis. Here is an example:


        res <- quid(id = stroop$ID, condition = stroop$cond, rt = stroop$rt)
      

The output, res, is a list containing the posterior-mean estimates from the unconstrained model for each individuals’ effect (*θ_i_*), the posterior overall effect (*μ*_θ_), the posterior standard deviation of estimated effects (*σ*_θ_), the Bayes factors comparisons among the three models, and raw outputs from the underlying MCMC chains. The Bayes factor comparisons are stored in res$bfs, which contains three elements. The first element, bf.1u, is the Bayes factor between the common-effect and the unconstrained model. It is near unity indicating equivalent evidence for each. The second element, bf.pu, is the Bayes factor between the positive and unconstrained model, and it is 6.43 indicating moderate evidence for only quantitative individual differences.^6^ Moreover, we can obtain the Bayes factor between the positive and common-effect model by division. It is bf.pu/bf.1u, which in this case evaluate to 6.94. Individual posterior mean effect estimates are provided in the vector res$ind.effects. ***Figure 2A*** was drawn by sorting these effects from smallest to largest.

One input missing from the above function call is the prior settings. There are two critical values: (a) about how large we expect the mean effect to be, and (b) about how much we expect individuals to differ from this mean effect. The exact details of what these settings mean is provided in Haaf & Rouder (2017), but even without these details, choosing values for these settings is relatively straightforward. The first concept we need is a sense of how variable repeated trials are. In a response time experiment with subsecond responses, the standard deviation within a person and task is somewhere between 150 ms to 250 ms. Next, we need some sense of how large the effect might be, and in typical RT experiments it is about 50 ms. The first setting in the prior is the ratio—how big is the effect relative to trial noise. Reasonable settings range from about 1/4 to 1/7, and the default setting is 1/6. The next setting is how variable people are around this mean effect. We tend to think that the standard deviation of true individual effects is a bit smaller than the mean effect, say from 20 ms to 40 ms or so in RT experiments. When expressed as a ratio of the trial noise, values that range from 1/11 to 1/5 seem appropriate, and the default setting is 1/10. These values may be changed easily. For example, if we think trial noise is about 200 ms, and the effect is large, say 80 ms, and we think people vary quite a lot, say 40 ms, we could set the priors as follows:


        largeVals <-c(80/200, 40/200)
resB <- quid(id = stroop$ID, condition = stroop$cond, rt = stroop$rt,prior = largeVals)
      

The new Bayes factors are as follows: the positive model remains most supported though the common-effect model for these settings becomes more viable (*B_p_*_1_ = 1.68). The evidence remains against qualitative individual differences (*B_pu_* = 4.66). The increased attractiveness of the common-effect model makes sense—with prior specifications that anticipate larger individual differences, the observed small individual differences are more interstitial between no individual differences and the anticipated larger ones.

The fact that the Bayes factor model comparisons is sensitive to prior specification is sometimes critiqued (Aitkin, 1991; Gelman, Carlin, Stern, & Rubin, 2004; Kruschke, 2013; Spiegelhalter, Best, Carlin, & van der Linde, 2002). Yet, we along with many others, see the priors as important elements of model specification where researchers add theoretical and substantive constraint (Rouder, Morey, & Wagenmakers, 2016; Vanpaemel, 2010; Vanpaemel & Lee, 2012). In this view, the priors are part of the model, and methods that do not account for the priors in model comparison are insensitive to model specification. Rather than running away from subjective elements in the prior, researchers should treat them as hypotheticals used to query structure. For example, by changing the prior above, we gain confidence that the lack of qualitative individual differences may be demonstrated across a range of *reasonable* prior settings. Understanding this range is important context for understanding the inference.

The above demonstration made use of relatively common knowledge about RTs: for simple tasks such as the Stroop, they are usually less than a second, consequently, the standard deviation across repeated trials is somewhere around 1/4 second, and the like. We think similar knowledge exists for most dependent measures in cognitive psychology. For example, accuracy effects are on the scale of 10%; Likert effects are on the scale of 1/2 point on a seven point scale; and event-related potentials are on the scale of a few millivolts. By using this common knowledge, researchers can construct sets of priors that span a reasonable range and see how robust their inferences are to changes in these priors. An example of such prior bracketing is provided in much of our recent work (e.g., Haaf & Rouder, 2017, 2019; Rouder et al., 2019a).

## Analysis of an Orientation Effect

In this section, we work an example from Rouder, Yue, Speckman, Pratte, & Province (2010). The experiment was a simple orientation discrimination task—gabor gratings were tilted slightly left and slightly right of vertical, and the participant had to indicate the direction of tilt as either left or right. The tilts could be either 1.5°, 2.0°, or 4.0° from vertical. The degree of tilt serves as a strength variable, and performance was quickest to the 4.0° tilt and slowest to the 1.5° tilt. Even so, all tilts were large enough for accurate performance.

**Figure 4:**
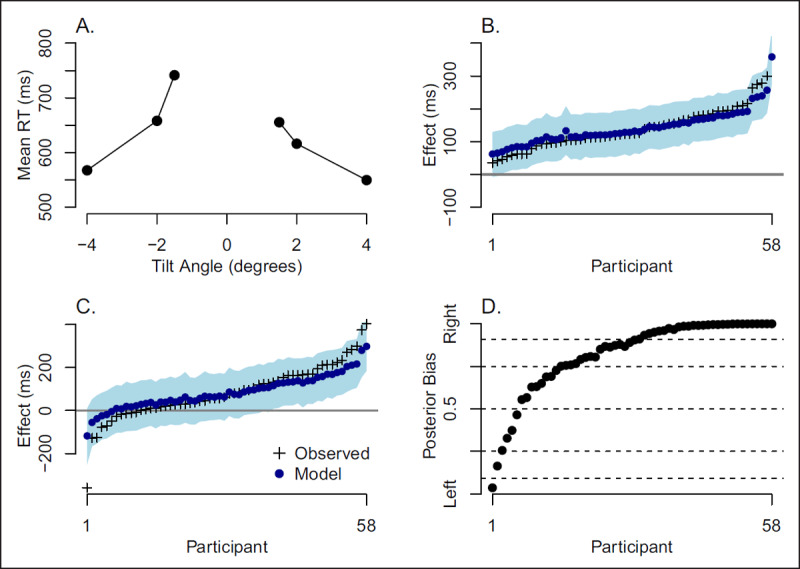
Analyses of orientation strength and bias effects. The data are from Rouder et al. (2010). **A.** Mean response time for correct responses as a function of tilt angle. Both strength and bias effects may be observed. **B.** Empirical and model estimates of individual strength effects. The evidence favors quantitative individual differences. **C.** Empirical and model estimates of individual bias effects. Although there is a significant overall rightward bias, the evidence here favors qualitative individual differences. **D.** Posterior probability that each participant has a rightward bias. Horizontal lines are for 1-to-1, 3-to-1, and 10-to-1 odds.

We use the data to assess whether there are qualitative individual differences in measures of strength and bias, which are defined as follows: The mean response time for correct responses to all the tilts is shown in ***Figure 4A***. The strength effect refers to the relative speed up for stimuli with more extreme tilts. For the purposes of this paper, the two most extreme tilts (–4° and 4°) define the strong condition, and the two most centered tilts (–1.5° and 1.5°) define the weak condition. The strength effect is simply the difference in response time between the weak and strong conditions. The question is whether each individual displays a positive true strength effect or whether there are individuals who have a true reverse strength effect.

***Figure 4A*** shows a bias effect, too. Responses to left tilts are slower than those to right tilts. Moreover, the bias appears disproportionately larger for the most centered tilts. Rouder et al. (2010) noted this bias and speculated that perhaps it was an encoding bias more than a handedness effect as it depended critically on the strength of the tilt. Here, we ask whether all people have a true rightward bias or are there individuals that have a true leftward bias. We use the difference between response times in the –1.5° and 1.5° conditions to define the bias effect. This difference averages 85 ms, which is a significant effect (t = 8.21, p < .05).

The analyses of the strength and bias effects were performed with separate calls quid() function. The following code loads the data from Rouder et al. (2010), cleans it according to the original authors’ specifications, and runs the strength analysis.


        source(’https://bit.ly/32Yvlel’)
dat=loadOrientationData()
strength=codeStrength(dat)
res.s=quid(id=strength$sub,
                    condition=strength$cond,
                    rt=strength$rt,
                    prior=c(.4,.4))
bias=codeBias(dat)
res.b=quid(id=bias$sub,
                    condition=bias$cond,
                    rt=bias$rt,
                    prior=c(.2,.1))
      

***Figure 4B*** shows the empirical mean effect, model estimates and model 95% credible intervals for the strength effect. The empirical mean effects are provided in the $ind.effect field. The model estimates (posterior mean) and model 95% posterior credible intervals are computed from the $theta field, the posterior samples for each individual. There is not much shrinkage in ***Figure 4B*** because there are many trials per individual in the design.

The function quid() provides Bayes factors. We used larger prior settings then in the previous example. Here, the experiment was designed to yield large effects so that the shape of the distributions could be assessed. From pretesting, we expected over a 100 ms effect on 250 ms of trial noise. The ratio here is .4, which we used above. We let the individual variation be this large as well. With these settings, the resulting Bayes factors for the strength effect indicate that the positive model is preferred to the unconstrained model by about 8-to-1, and is vastly superior to the common effect model (10^9^-to-1) or the null model (10^152^-to-1). It seems, perhaps not too surprisingly, that everyone has a positive orientation strength effect where responses are quicker to larger tilts.

Perhaps the more interesting case is the bias effect (***Figure 4C***). Here, the plot shows an overall rightward bias, which is highly significant by the usual *t*-test. Yet, from inspection it seems that some individuals have a leftward bias. Indeed, the Bayes factors confirm this observation. The unconstrained model is preferred to all three competitors. It beats the common-effect model by about 10^10^-to-1 and null model by about 10^27^-to-1. It also beats the positive model with Bayes factor of about 1700-to-1 in favor of the unconstrained model.

The overwhelming evidence for the unconstrained model is strong support for qualitative individual differences in this bias effect. While the majority have a rightward bias where slight rightward tilts are discriminated faster than slight leftward tilts, some individuals have a true reversal. ***Figure 4D*** shows the posterior probability that a participant has a rightward bias. These probabilities are computed from the $theta field in the quid() function. The probability estimate is simply the proportion of posterior samples that are positive. From ***Figure 4D***, 6 of 58 participants (10.3%) are more likely to have a leftward than rightward bias. The horizontal lines show cutoffs for 3-to-1 and 10-to-1 odds. With these more stringent criteria, 2 participants (3.5%) meet the 3-to-1 criteria for leftward bias and 1 participant (1.7%) meets the 10-to-1 criteria for leftward bias. We think the presence of individuals with leftward and rightward bias motivates questions that extend beyond the mean. Who are these leftward-bias individuals, and what are the correlates of leftward bias?

## Sample Size Considerations

The study of individual differences entails different experimental design and sample size considerations than the study of population averages. One needs much more data, and in particular, one needs many more trials per person per condition. ***Figure 2*** is useful here. ***Figure 2A*** comes from about 50 trials, ***Figure 2B*** comes from about 100 trials per person and condition. The sample effects in 2A are somewhat noisier because they are the differences of condition means over fewer trials.

Accurately identifying true individual differences demands increased resolution that may be satisfied only by using large numbers of trials per individual per condition. If high resolution is not achieved, then it will be difficult to observe any individual differences much less qualitative ones. When individual differences are small relative to the resolution of the data, then the models without individual differences, the common-effect model and the null model, may be preferred. If the common-effect models is preferred, we tend to think of it more as a comment on the resolution of the design—the design had sufficient resolution to resolve the overall effect but insufficient resolution to resolve individual differences.

How many trials are needed to resolve individual differences? The number of needed trials will depend on how much trial noise there is. But a good rule of thumb is that 100 trials per person per condition are needed, and the ability to see differences increases with even further increasing trial sizes. The bottom line is that when exploring individual differences, the number of trials is as important if not more than the number of individuals.

## A Topography of Qualitative Individual Differences

With the above models and tools, researchers are well positioned to ask if there exist qualitative individual differences on a task or in a domain.

### No Qualitative Individual Differences

When we started thinking about qualitative individual differences, it seemed easy to think of tasks and conditions where there were likely none. Here are some cases where we think qualitative individual differences do not exist.

#### Unidimensional Physical Strength Variables

In many domains, the stimuli vary on a single physical dimension. Examples include the detection of faint tones of varying loudness and the identification of briefly flashed masked letters. In the latter, the duration of the brief flash serves as the dimension on which stimuli vary. There are stimulus-strength dimensions in memory and linguistics as well, for example how often a memoranda is repeated or the frequency-of-occurrence of words. We suspect that tasks with stimuli that vary on a single physical dimension admit no qualitative individual differences. Indeed, it would be shocking if anyone responds more quickly to soft than loud tones, identifies letters of shorter durations better than longer ones, recalls oft-presented memoranda worse than once-presented memoranda, or names rare words more quickly than common ones.

#### Context and Priming Effects

We also suspect that there are no qualitative individual differences in most priming and context effects. Take, for example, the Stroop effect—it is highly plausible that all people truly respond more quickly to congruent than incongruent items. The same plausibility holds for most context effects including flanker effects, Simon effects, word congruency effects and the like. Moreover, there are context effects that are not conflict based, say the enhancement of letter identification when the letter is placed within a word (Neisser, 1967; Reicher, 1969; Rumelhart & Siple, 1974). As speculation, it seems likely that these types of effects are universal as well.

### Qualitative Individual Differences

Here are some cases where we think there will be qualitative individual differences.

#### Laterality

One set of tasks where there are obvious qualitative individual differences is laterality. When defined with reference to a task, say throwing a ball, left-handedness is obviously a qualitatively different than right-handedness. Moreover, left-handedness seems to correspond to many phenomena including shorter life spans, greater susceptibility to psychopathology, and increased skills in mathematics and visualization (Coren, 1993).

#### Preference

Tasks where people express preferences admit qualitative individual differences. For example, we may present people with two beverages, say unsweetened and sweetened ice tea and ask them to move a slider expressing their preference. At one pole is the sweetened tea, and at the other is the unsweetened tea. Some people will express a preference for one, others for the other, and still others may be equivocal between them. This is not to say that all preference domains admit qualitative individual differences. Surely, we can construct preferences that do not admit them, say winning the lottery vs. losing the lottery. But, when the preference is about aesthetics, taste, values, and politics, of course people will differ qualitatively.

In the study of human decision making one of the goals is to reduce the seemingly arbitrary qualitative differences of preference to quantitative ones. The notion is that perhaps all decision makers go through the same processes at combining and evaluating information with the only differences being quantitative differences in utilities and weights (Resnik, 1987). A critical question is the singularity of such processes. Many researchers studying decision making address this question by categorizing individuals as following one decision-making strategy or another, for example, whether people follow lexicographic or transitive structures in combining features (Davis-Stober & Brown, 2011; Regenwetter et al., 2008). Yet, the act of categorizing is not a principled approach to establishing qualitative individual differences (see Thiele, Haaf, & Rouder, 2017 for an elaborated critique). Decision-making researchers may be better served by the developments herein to address reducible qualitative individual differences in decision making strategies.

##### Biases

In the provided example, we observed an orientation bias where most people displayed a rightward bias but some displayed a leftward bias. This finding raises the prospect that biases—settings that do not affect performance but do effect response patterns—may be qualitative. Here is a less obvious example. Schnuerch, Nadarevic, & Rouder (2020) examined individual differences in the *truth effect* with the methods outlined here. The truth effect occurs when people rate statements as more likely to be true when they are repeated. This is a bias of sorts, and it probably is used on statements where the participant is guessing about the truth of the statement. Schnuerch et al. (2020) found that wherever individual differences were resolvable the unconstrained model outperformed the positive model. Their conclusion was that while most people had a true truth effect, a minority had the opposite. This minority truly discounted the truth of repeated statements. And Schnuerch et al. (2020) further concluded that it was unlikely that the mental processes that gave rise to the truth effect were the same as those that gave rise to its reversal. Hence, the effect of repetition was complex and varied, and most likely dependent on additional factors such as how conscious people are of previously encountering the item (Nadarevic & Erdfelder, 2014).

##### Multiple-Process Accounts

Many psychological theories imply that there should be qualitative individual differences. Take, for example, the dual-process memory theory that emphasizes the separate roles of fast, automatic familiarity processes and slower, more deliberative recollective ones (Chaiken & Trope, 1999; Yonelinas, 2002). Here, it is reasonable to assume that some people rely more on one process than the other as a matter of rather ordinary individual differences. Indeed, one related covariate is aging, and elderly adults tend to rely on familiarity to a greater degree than young adults (Jennings & Jacoby, 1993). Hence, we should experimentally be able to put familiarity and recollection in conflict and expect qualitative individual differences. For example, consider a directed forgetting experiment where a word-pair has greatly amplified familiarity. We might expect those with poor recollection to endorse this pair as old perhaps at a greater rate than they would target old items. Those with good recollection may do the opposite. According to the theory, much as we can engineer sound streams that give rise to qualitatively different interpretations, we should be able to engineer conflict experiments that yield qualitative individual differences. If we cannot, then perhaps we should worry about the falsifiability of the dual-process claim.

## General Discussion

In this paper, we promote a new set of questions for cognitive inquiry that focus on individuals rather than averages. Our main target is the question of qualitative individual differences—does everybody have an effect in the same direction or is there variability in the direction of effects. We believe that in some domains there are no qualitative individual differences (say strength effects), and in others there are irreducible qualitative individual differences (say laterality). Perhaps most exciting is a third class of domains—those with reducible qualitative individual differences where these differences may be explained by quantitative individual differences in rich processing mechanism. In this regard, studying the conditions under which there are and are not qualitative individual differences may serve as an important way of providing a phenomenological basis for advanced theory building.

While we can make a case that these new questions provide an enhanced view of phenomena, they have had little impact. We can think of lots of reasons why this is so. First, it may be that these new questions about qualitative individual differences do not interface well with existing theory of processing. Most of our theories reflect experimental manipulations, and by focusing on the configuration of individual differences, we have moved the focused from manipulated variables to observed variation. Second, it may be that the answer to these new questions in many cases is fairly obvious, for example with strength stimuli. Third, studying qualitative individual differences requires large studies with carefully chosen stimuli. Setting up and executing these experiments may present a pragmatic hurdle. Fourth, researchers may find these new hierarchical modeling methods daunting. Hopefully, the software we highlight here will help those who would like to try.

In the end, we think there is unrealized promise in the study of qualitative individual differences. We would like to hear if you find this focus useful and, if so, in what contexts. And if not, why not?

## Data Accessibility Statement

All data reanalyzed in this article are publicly available. The Stroop data of ***Figure 2*** are available at Rouder et al. (2019); the orientation-judgment of ***Figure 4*** are available at ***raw.githubusercontent.com/PerceptionCognitionLab/ctx-inhibition/public/shared/rouder.2010.orientation.dat***.

## Footnotes

A good example is the show *My Strange Addiction*. See ***https://en.wikipedia.org/wiki/My_Strange_Addiction***.

See ***https://en.wikipedia.org/wiki/The_dress*** and ***https://en.wikipedia.org/wiki/Yanny_or_Laurel*** for illustration.

A far more humorous version of this effect was inadvertently created by the Sesame Street character Grover who may be perceived to use offensive language with Rosie. Grover says “That sounds like an excellent idea,” but many perceive him as saying “That’s a fucking excellent idea” (see ***https://www.youtube.com/watch?v=va2rJicOPYk***).

The full specification of the model may be found in Haaf & Rouder (2017). Here we provide an overview sketch of the main dynamics.

***https://github.com/jstbcs/play/blob/master/Qualitative%20Individual%20Differences/example.Rmd***.

Oh Snap! In Haaf & Rouder (2017), we report the same Bayes factors with the same models on the same data as 12 rather than 6.43. Why? Because there was an inadvertent error in Haaf & Rouder (2017) that led to an overstatement of these Bayes factors. The values here are correct.
